# Canine Epithelial Skin Tumours: Expression of the Stem Cell Markers Lgr5, Lgr6 and Sox9 in Light of New Cancer Stem Cell Theories

**DOI:** 10.3390/vetsci7020062

**Published:** 2020-05-08

**Authors:** Laura Bongiovanni, Chiara Brachelente, Eva Moreno, Monika M. Welle

**Affiliations:** 1 Department of Biomolecular Health Sciences, Faculty of Veterinary Medicine, Utrecht University, 3584 CT Utrecht, The Netherlands; E.Moreno@uu.nl; 2 Faculty of Veterinary Medicine, University of Teramo, 64100 Teramo, Italy; 3 Department of Veterinary Medicine, University of Perugia, 06123 Perugia, Italy; chiara.brachelente@unipg.it; 4 Institute of Animal Pathology (ITPA), Vetsuisse Faculty, University of Bern, 3012 Bern, Switzerland; monika.welle@vetsuisse.unibe.ch

**Keywords:** cancer, dog, hair follicle, immunohistochemistry, qRT-PCR, skin, stem cell marker, tumour-initiating cell

## Abstract

Evidence is accumulating that tumour development is driven by cancer stem cells (CSCs). In order to understand the presence and potential contribution of stem cells (SCs) as tumour-initiating cells in canine cutaneous tumours, we selected three putative SC markers (Lgr5, Lgr6 and Sox9) and investigated their expression pattern, level of protein and mRNA expression, in 43 canine hair follicle (HF) and 18 canine cutaneous epidermal tumours by immunohistochemistry and qRT-PCR, using normal skin samples as controls. Lgr5 protein expression was not detected in epidermal and HF tumours; however, *Lgr5* mRNA overexpression was evident in some HF tumours. Sox9 was expressed in several tumour cases, both at the protein and mRNA level. The Lgr6 antibody tested was not suitable for formalin-fixed paraffin-embedded tissue samples, but *Lgr6* gene showed higher expression in several samples of both HF and epidermal tumours compared with normal skin. Significantly higher mRNA expression levels of the three SC markers were found in trichoblastomas (TB) compared with basal cell carcinomas (BCC). The present results indicated that canine HF and epidermal tumours might have common tumour-initiating cells. The mRNA expression of the three selected SC markers, especially Lgr5, could be potentially useful in the distinction between canine TB and BCC.

## 1. Introduction

A hierarchical organization of tumour cells has been proposed for most of the tumour types, based on the existence of a specific population of tumour-initiating cells (TICs) with unlimited capacity for self-renewal and proliferation. In the so-called TIC model, a specific neoplastic cell population exhibits stem cell characteristics, having the ability to generate both, differentiated, highly proliferative tumour cells as well as other self-renewing TICs that can regenerate all tumour cell types [1]. The term TICs is often used interchangeably with “cancer stem cells” (CSCs). Recent findings demonstrated that CSCs do not necessarily have to be rare and/or quiescent, but they can be abundant and proliferate actively. A new concept has emerged in the last years about the active role of the stem cell niche in regulating the hierarchical organization of tumour cells. Proliferation and quiescence of CSCs, like in normal stem cells, are determined by signals derived from their niche, while the size of the niche itself determines the number of stem cells [2]. Only cells that remain within the niche are stem cells, while those that leave the niche undergo differentiation into transient amplifying (TA) cells which in turn undergo final differentiation. However, both the TA cells and differentiated cells can be reprogrammed into stem cells by niche signals [2]. Interestingly, an inverse correlation between niche dependency and malignancy has been observed. Tumour progression occurs through the acquisition of genetic alterations; as a consequence, genetic variants render CSCs progressively independent of niche signals. The mutated, independent CSC phenotype impedes differentiation, changing the tumour population with many undifferentiated CSCs and a few non-CSCs [2]. This new CSC concept is in line with findings indicating a correlation between the expression of stem cell (SC) markers and tumour progression, malignancy and poor prognosis in many human and canine cancer types [3,4]. Indeed, CSCs/TICs express stem cell markers, overexpress multidrug resistance molecules [5] and share several characteristics with normal, adult SCs.

Permanently residing adult stem cells have been recognized in normal skin, and these are able to sustain three principal differentiated lineages: the interfollicular epidermis (IFE), the sebaceous glands (SGs) and the hair follicles (HFs), thus maintaining skin structure and integrity [6]. In canine skin, several stem cell markers, including CD34, CK15, follistatin, CD200, Lgr5, Lgr6 and Sox9 [7,8,9,10,11,12], have been investigated and demonstrated to have similar expression profiles as *human* and *mouse* HF SC markers.

The exploration of biomarkers of canine skin cancer progression has been a long-standing focus of our research group, both with the purpose of better understanding the biology of these tumours and finding new potential therapeutic targets. We have previously demonstrated the overexpression of several molecules known to be markers of TICs in canine epithelial skin tumours, such as β-catenin and heat shock proteins (HSPs) [13,14], as well as the overexpression of several stem cells markers [15,16]. These findings suggest a potential implication of these molecules in the development, maintenance and/or progression of canine skin tumours. A few other published studies focused on the examination of selected putative stem cell markers to better classify canine cutaneous epithelial tumours and their cell of origin [17,18]. However, the exact role of these cells as TICs in canine skin tumour development and progression is not understood. 

Based on a literature review, we selected three putative HF stem cell markers that have been suggested as CSC markers in several tumour types, including skin tumours. The leucine-rich repeat-containing G-protein-coupled receptors Lgr5 and Lgr6 are receptors involved in the Wnt signalling pathway that have been identified as markers of stem cells in various tissues including the intestine and the hair follicle [19]. Lgr5 marks actively cycling stem cells (SCs) and a multipotent population in the hair follicle (HF). It maintains the cycling part of the HF and thus contributes to the formation of all HF structures [20]. Lgr6 is a marker for distinct stem cells and is able to give rise to all lineages of the skin (HF, sebaceous gland and interfollicular epidermis) [21]. Sox9 is a transcription factor expressed in the sebaceous and sweat glands and the outer root sheath of the HF, as well as in the bulge [22].

The aim of the present work was to evaluate the presence, immunostaining pattern and mRNA expression level of selected putative stem cell markers (Sox9, Lgr5 and Lgr6) in a set of canine cutaneous epithelial tumours. The investigated tumours included hair follicle tumours (trichoblastomas (TB), trichoepitheliomas (TE), pilomatricomas (PM), infundibular keratinizing acanthomas (IKA), trichilemmoma (TL)) and epidermal tumours (squamous cell carcinomas (SCC) and basal cell carcinomas (BCC)). We expect that the result of this study on stem cell marker expression and localization may aid to understand the contribution of TICs/CSCs in the development, maintenance and progression of canine skin cancers. Since the selected SC markers are markers of canine hair follicle stem cells [11] and are not expressed in the interfollicular epidermis in healthy skin conditions, the present work also aimed to investigate whether a possible common cell of origin, residing within the HF stem cell niche, contributes to the development of canine epidermal tumours (SCC and BCC). 

## 2. Material and Methods

### 2.1. Tumour Samples 

Archival tissue samples of cutaneous epithelial tumours submitted to the biopsy service of the Institute of Veterinary Pathology, Vetsuisse Faculty of the University of Bern, were used for this study. Selection criteria were a definite histological diagnosis and good preservation of the samples. A total of 52 canine skin tumours were selected and consisted of 37 hair follicle tumours, including benign (8 TB, 5 TE, 4 PM, 7 IKA and 3 TL) and malignant (6 TE and 2 PM) forms, and 15 canine malignant epidermal tumours (9 SCC and 6 BCC). As internal control, 3 samples of normal skin surrounding, but distant to, the tumour mass were evaluated immunohistochemically and processed separately for RNA isolation and subsequent qPCR analysis. 

### 2.2. Histological Examination

All specimens were fixed in 10% neutral buffered formalin, embedded in paraffin, sectioned at 4–5 μm, stained with haematoxylin and eosin (H&E) and examined by light microscopy (Leica, DL-MS, Wetzlar, Germany). Tumours were classified according to the World Health Organization criteria [23] for canine cutaneous epithelial tumours and according to the classification of Gross et al. [24]. For each sample, the growth pattern (expansive or infiltrative growth), presence of necrosis, ulceration, cellular/nuclear pleomorphism, inflammatory (lymphocytic/lymphoplasmacytic) infiltration and the number of mitotic figures were evaluated as additional features. Malignancy was assessed considering the mitotic index, cellular/nuclear pleomorphism, and infiltrative growth. Mitoses were evaluated by counting the number of mitotic figures in 10 random, non-overlapping fields at the highest magnification (HPF, 40×) in H&E stained slides. 

### 2.3. Immunohistochemistry (IHC)

Sections were deparaffinized and rehydrated by passage through xylene and graded ethanol. Immunohistochemistry was achieved using the following antibodies: rabbit polyclonal Lgr5 (HPA012530, Sigma-Aldrich, 1:1200, St. Louis, MO, USA), rabbit polyclonal Lgr6 (sc-99123, Santa Cruz Biotechnology, different dilutions were tested from 1:50 to 1:1000, Dallas, TX, USA) and rabbit polyclonal Sox9 (sc-20095, Santa Cruz Biotechnology, 1:250, Dallas, TX, USA). Antigen retrieval was carried out in either sodium citrate buffer (self-made), pH 6.0, for 20 min at 80 °C (Lgr5) or in Tris-EDTA buffer (self-made), pH 9.0, for 15 min in a pressure cooker (Sox9). Non-specific background was blocked using 5% dried skim milk in phosphate-buffer saline (PBS). Subsequently, primary antibodies were diluted in Dako REAL^TM^ Antibody Diluent (S2022, Dako, Baar, Switzerland) and applied for 60 min at room temperature (Lgr5) or overnight at 4 °C (Sox9). The stained sections were further processed according to the manufacturer’s instructions of EnVision+ Kits (Dako). 3-Amino-9-ethylcarbazole (AEC) was applied as the chromogen (5–10 min). Tris-buffered saline (TBS; pH 7.6) (Sox9) or PBS (Lgr5) (self-made) was used for the washing steps. All sections were counterstained with Ehrlich’s haematoxylin. Separate sections were also incubated with equal concentrations of normal rabbit IgG (sc-2027, Santa Cruz Biotechnology, Dallas, TX, USA) as negative controls. Immunohistochemical protocols for Lgr5 and Sox9 were adapted from Gerhards et al. [11] and normal skin samples were used as positive controls. For Lgr6 (sc-99123, Santa Cruz Biotechnology, Dallas, TX, USA), we performed several experiments using both immunohistochemical and immunofluorescence techniques (as reported in Supplementary File 1). As positive controls, human normal skin as well as canine trichoblastoma cases (where a high mRNA expression level for *Lgr6* was observed) were included.

### 2.4. Quantification of Immunolabelling 

A semiquantitative immunohistochemical assessment was performed, considering nuclear immunostaining in the entire tumour tissue (cross-section) for each case. Samples were subdivided based on the protein expression levels in five ranges: absent, no positive cells; low, >0% to <10% positive cells; moderate, ≥10% to <25% positive cells; high, ≥25% to <50% positive cells; very high, ≥50% positive cells.

### 2.5. RNA Isolation and cDNA Synthesis

Three 20-μm-thick sections of each case were used for RNA extraction. Prior to RNA extraction, the surrounding normal skin was cut off in order to ensure that only the gene expression of tumour tissue is investigated. Three samples of normal skin were used as controls.

RNA extraction was performed according to the instructions for the use of the RNeasy Fibrous Tissue Mini Kit (74704, Qiagen, Hombrechtikon, Switzerland). Concentrations were assessed using a NanoDrop ND-1000 spectrophotometer. The synthesis of cDNA was carried out with reagents from Promega AG (Dübendorf, Switzerland). In detail, the mixture contained 2 µL M-MLV Reverse Transcriptase (M3682, Promega), 10 µL 5× Reaction Buffer, 2 µL RNasin Ribonuclease Inhibitor (N2115, Promega), 2.5 µL dNTP Mix (U1515, Promega), 2.5 µL Random Primers (C1181, Promega), 11 µL H_2_O and 2 µg RNA dissolved in 20 µL H_2_O. Incubation was started at 25 °C for 10 min, followed by 60 min at 42 °C, and was finished after 10 min at 95 °C. The cDNA was stored at –20 °C until use.

### 2.6. Quantitative Real-Time PCR

The amplification mixture contained 12.5 µL TaqMan Universal PCR Master Mix (4304437, Applied Biosystems, Life Technologies, Zug, Switzerland), 2 µL of cDNA and 2.5 µL of 10 µM primers and probes in a final reaction volume of 25 µL by the addition of RNase-free water. Primers are listed in Table 1. Quantitative PCR was performed for 50 repeats on AB 7500 Fast Real-Time PCR System (Applied Biosystems, Applied Biosystems, Life Technologies, Zug, Switzerland). Gene expression was normalized to the level of ribosomal 18S and calculated based on the ΔΔCT method [25]. Reactions were performed in triplicates and fold change expression was calculated for each gene using normal skin as the reference.

### 2.7. Statistical Analysis 

Fisher’s exact test was applied to evaluate the significance of the protein (Sox9) expression levels and their correlation with histopathologically assessed malignancy. For this purpose, the cases were grouped as follows: 0 (absent) versus >0 positive cells (from low to very high semiquantitative evaluation). ANOVA tests were applied to compare the mRNA expression level of each marker in the epidermal tumours with that of normal skin, as well as the HF tumours and normal skin. A two-tailed t-test was used to compare the mRNA expression levels between BCC and TB, and between benign and malignant TE cases. The conventional 5% level was used to define statistical significance. 

## 3. Results

All results, both from immunohistochemistry and RT-qPCR, are summarized in Supplementary Table S2. 

### 3.1. Stem Cell Marker Analyses in Healthy Skin. Sox9 and Lgr5 Protein Expression was Confirmed in Specific Regions of Canine Hair Follicle

The presence of the mRNA of the three selected SC markers in canine skin was confirmed using RT-qPCR. Ribosomal 18S was used as the reference gene.

Lgr6 protein was not detected immunohistochemically in normal control skin samples. Based on our experiments and on the fact that the same antibody was previously confirmed to work on fresh canine normal skin samples by western blot [11], we concluded that the applied anti-Lgr6 antibody was not working on formalin-fixed paraffin-embedded tissue samples. 

**Figure 1:**
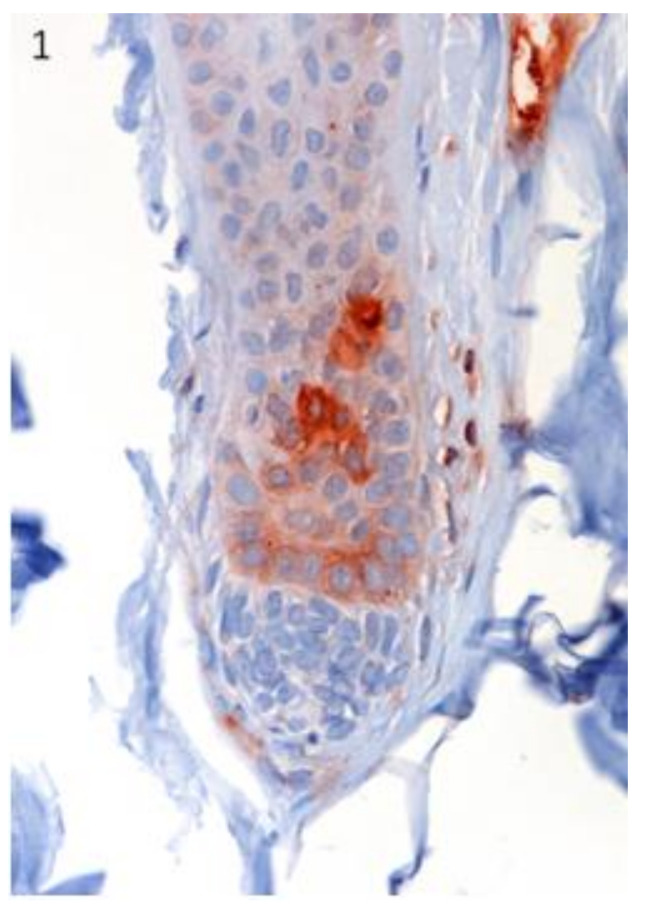
The localization of Lgr5 was confirmed in specific regions of canine hair follicle in healthy skin. Lgr5 protein expression was immunohistochemically evident in the secondary germ of the telogen and early anagen hair follicle (HF). Magnification 40×.

Lgr5 protein expression was immunohistochemically evident in the secondary germ of the telogen and early anagen HF (Figure 1). 

**Figure 2:**
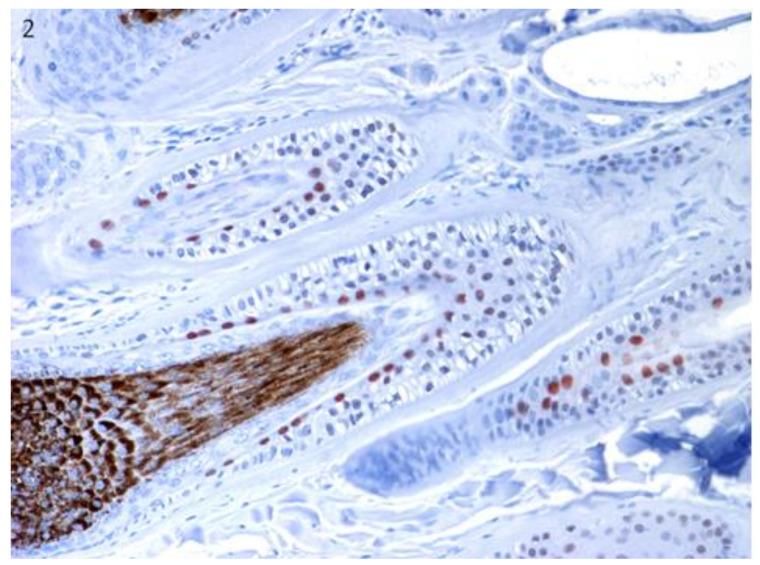
The localization of Sox9 was confirmed in specific regions of canine hair follicle in healthy skin. Sox9-positive cells in the innermost cell (IMC) layer of the outer root sheath (ORS) in the isthmus of late anagen hair follicles. Magnification 20×.

Sox9 immunostained cells were observed in the innermost cell (IMC) layer of the outer root sheath (ORS) in the isthmus of late anagen hair follicles (Figure 2). Both markers did not stain normal epidermis.

### 3.2. Stem Cell Marker Analyses in Epidermal Tumours. Lgr5 and Sox9 are Downregulated Compared with Normal Skin

**Figure 3:**
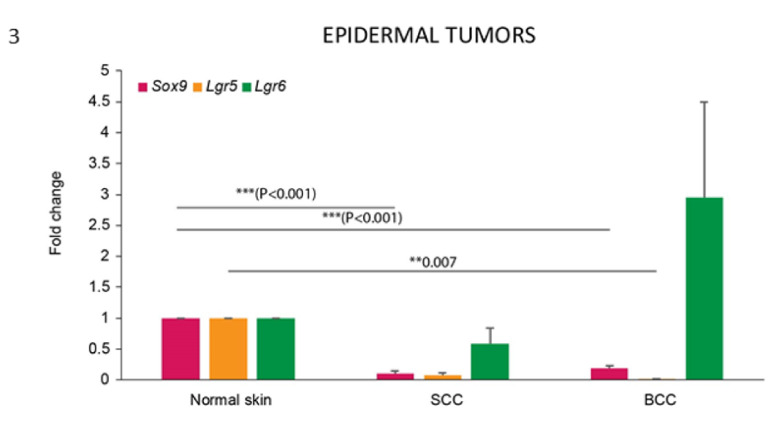
*Lgr5* and *Sox*9 are downregulated in epidermal tumours compared with normal skin. mRNA expression of *Sox9*, *Lgr5* and *Lgr6* in normal skin and epidermal tumours. Bars represent mean ± SEM. ** *p* < 0.05; *** *p* < 0.001. Normal skin tissues (normal skin, *n* = 3), basal cell carcinomas (BCC, *n* = 6), squamous cell carcinomas (SCC, *n* = 9).

*Lgr6* mRNA was detected in both BCC and SCC, and appeared to be upregulated in most of the BCC cases compared with normal skin and SCC. However, due to the high variation of the values, the differences were not statistically significant (Figure 3). 

In contrast, the mRNA expression of both *Lgr5* and *Sox9* was significantly downregulated in these tumours when compared with normal skin (Figure 3). 

*Lgr5* mRNA downregulation was statistically significant for the BCC samples, while protein expression was not observed in any of the tumours analysed, confirming Lgr5 as a more specific HF stem cell marker. 

**Figure 4:**
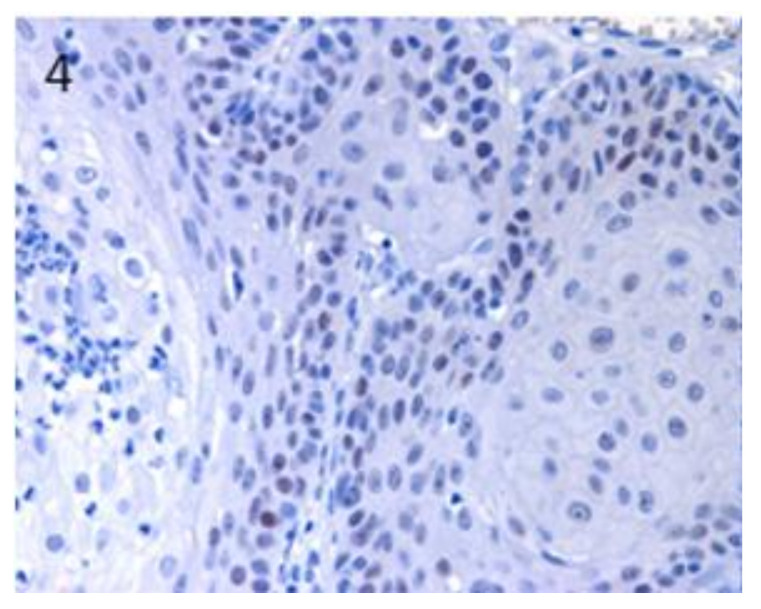
In SCC, Sox9-positive cells were mainly present in neoplastic cells showing basal cell morphology. Magnification 40×.

**Figure 5:**
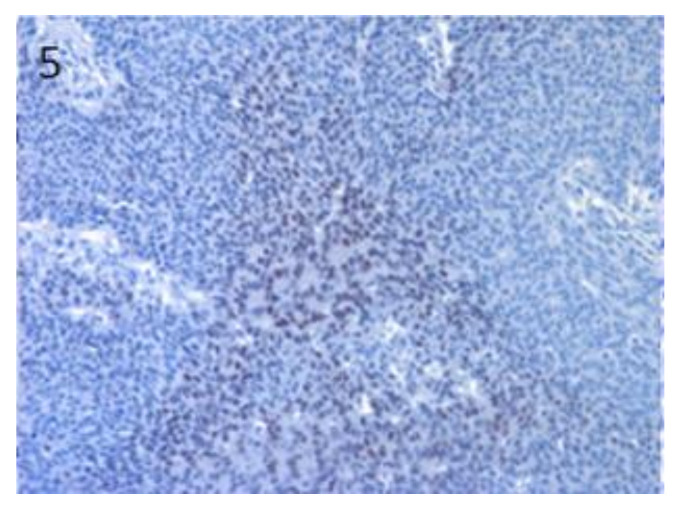
In basosquamous SCC, high Sox9 expression was observed in tumour cells with basal cell morphology, mainly in the inner parts of the neoplastic islands. Magnification 20×.

**Figure 6:**
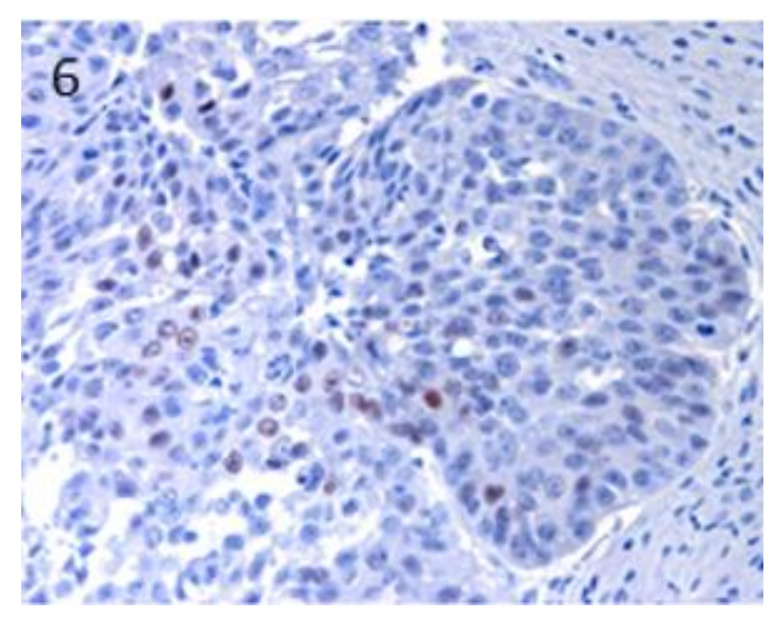
In BCC, a patchy distribution of immunostaining was observed. Magnification 40×.

*Sox9* mRNA expression was observed in both BCC and SCC, but it showed a significant downregulation compared with normal skin. When the pattern of distribution of the protein was analysed, about 50% of the cases of SCC (5/9) and BCC (3/6) showed positive cells (Table 1). In the stained SCC samples, Sox9-positive cells were mainly present in the neoplastic cells showing basal cell morphology (Figure 4), in infiltrating areas, and with a high level of expression (25%–50% of positive cells) in the specific SCC histotype known as basosquamous carcinoma (Figure 5). A patchy distribution of immunostaining was observed in the positive BCC cases (Figure 6).

### 3.3. Stem Cell Marker Analyses in Hair Follicle Tumours. TB Samples Show the Highest mRNA Expression of Lgr6, Lgr5 and Sox9, that are Potentially Useful Markers in Differential Diagnosis with BCC 

**Figure 7:**
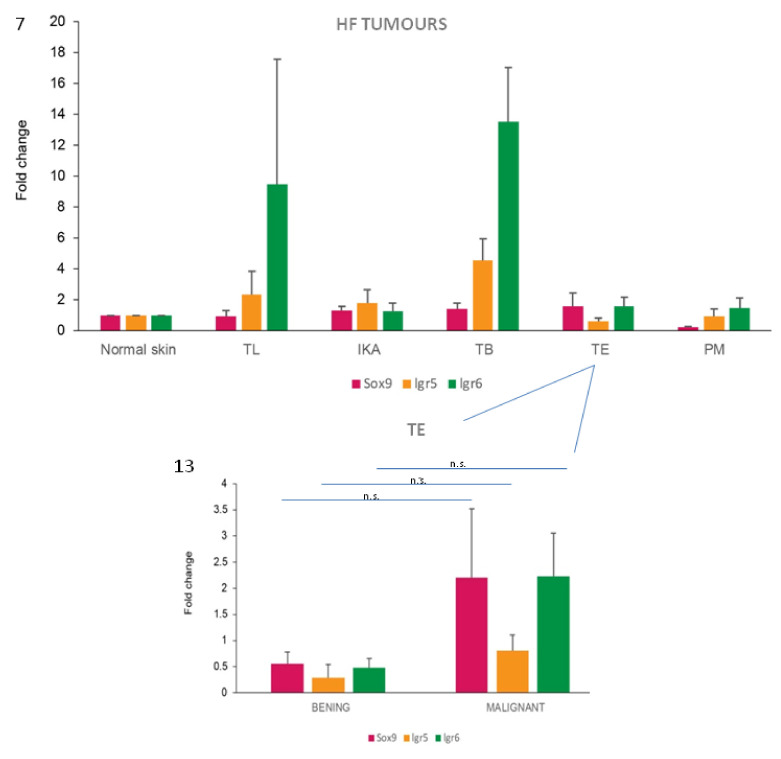
TB samples show the highest stem cell marker mRNA expression levels. mRNA expression of *Sox9, Lgr5* and *Lgr6* in normal skin and HF tumours. Bars represent mean ± SEM. No statistically significant differences were observed comparing each tumour with normal skin. Normal skin tissues (normal skin, *n* = 3), tricholemmomas (TL, *n* = 3), infundibular keratinizing acanthomas (IKA, *n* = 9), trichoblastomas (TB, *n* = 8), trichoepitheliomas (TE, *n* = 11), pilomatricomas (PM, *n* = 6).

*Lgr6* mRNA was detected in all the HF tumour types, with the highest expression values in TB and TL (inferior type). However, the expression varied significantly among the samples of the other HF tumour types, reaching occasionally similar levels to the ones expressed in TB and TL samples (Figure 7). 

*Lgr5* mRNA expression was observed in all the HF tumours, with the highest levels in TB cases, with an average fold change increase of 14 compared with normal skin. Very low mean levels of *Lgr5* gene expression, lower than that observed in normal skin, were recorded in TE and PM cases (Figure 7). Immunohistochemistry showed that Lgr5 protein was absent in all HF tumour samples analysed, despite the presence of positive immunostaining in the hair follicles of the skin surrounding the tumour mass.

*Sox9* mRNA expression was demonstrated in all tumour types analyzed, except for PM that showed very low/absent levels of expression when compared with values obtained from normal skin samples. The highest fold change was observed in TE and TB (Figure 7). The immunohistochemical expression of Sox9 was observed in all tumour types analyzed, but with a different percentage of positive cases and labelled neoplastic cells in the different groups (Table 1). 

**Figure 8:**
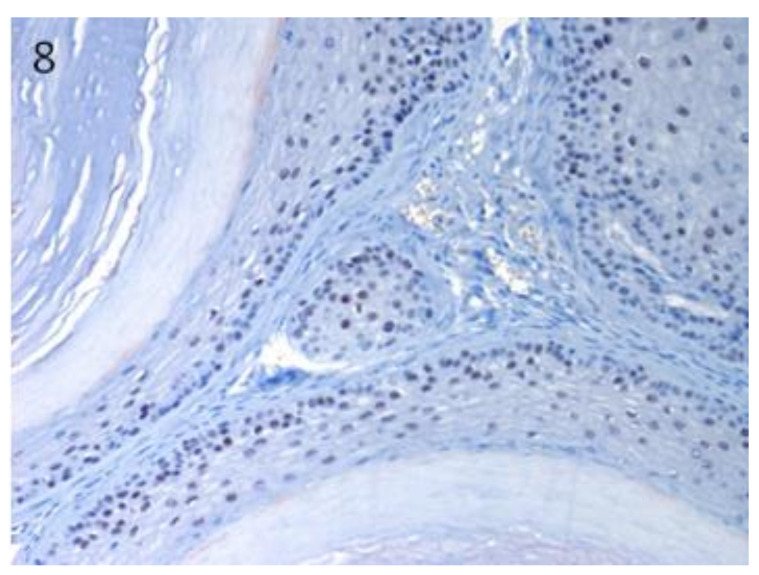
In IKA, Sox9-positive cells mainly present among the neoplastic cells of the basal cell layer of the tumour wall. Magnification 20×.

**Figure 9:**
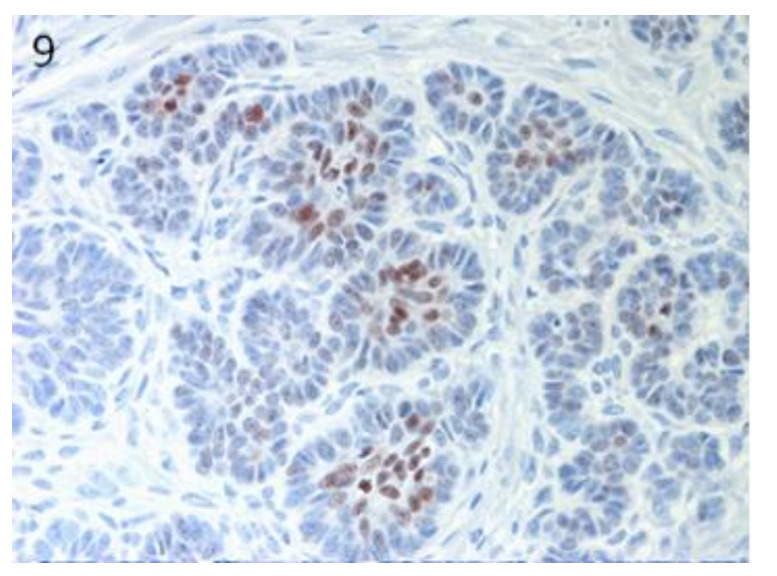
TB, ribbon type: positive neoplastic cells in the inner parts of the tumour nests. Magnification 40×.

**Figure 10:**
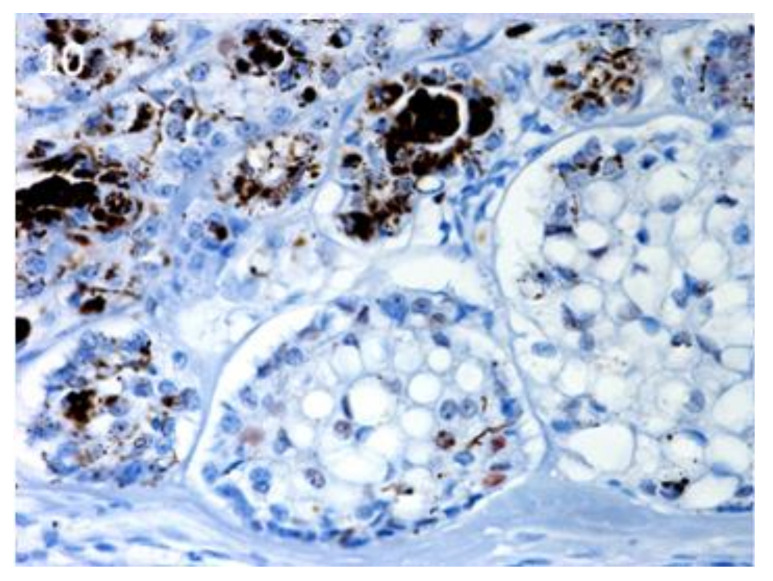
TL, inferior type: scattered, positive nuclei evident in the neoplastic islands. Magnification 40×.

**Figure 11:**
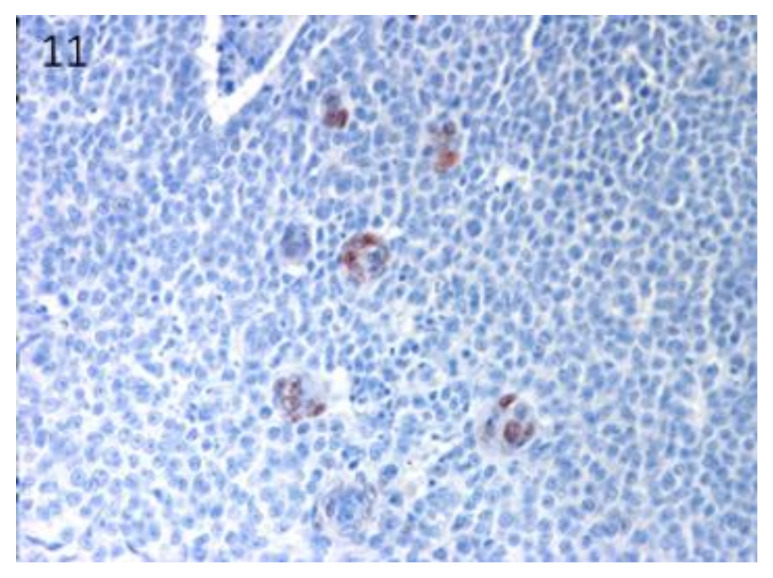
PM: negative neoplastic cells with matrical cell morphology associated with single or small clusters of positive cells. Magnification 40×.

**Figure 12:**
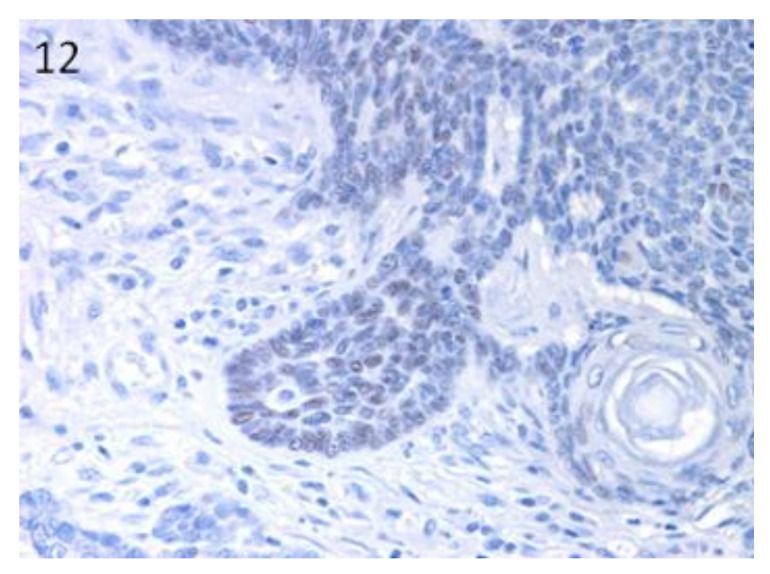
TE: groups of positive neoplastic cells within the parts of the tumour with basal cell morphology. Negative cells with squamous differentiation. Magnification 40×.

Sox9-positive cells were mainly present among the basaloid neoplastic cells of infundibular keratinizing acanthoma (Figure 8). The immunolabelling was evident in the inner parts of the nests of the TB, in both ribbon (Figure 9) and trabecular types, while only scattered positive nuclei were evident in the TL samples (Figure 10). In the PM cases, neoplastic cells with matrical cell morphology were negative; however, single or small clusters of positive cells were evident among these negative matrical cells (Figure 11). A heterogeneous expression pattern based on the predominant hair follicle differentiation was observed in TE samples (Figure 12), with no difference between the benign and malignant forms. 

When levels of mRNA expression of *Sox9, Lgr5* and *Lgr6* were compared between TB and BCC, statistically significant differences were observed for all the three markers, with higher expression levels in TB cases (*p* < 0.001).

**Figure 13:**
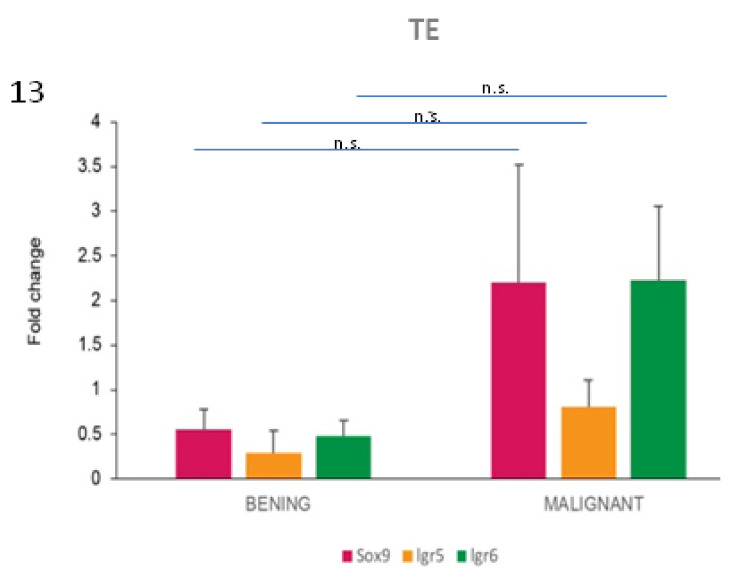
*Sox9* and *Lgr6* mRNA are potential markers of malignancy in TE tumours. mRNA expression of *Sox9, Lgr5* and *Lgr6* in TE cases. When the average values of benign and malignant cases were compared within the TE group, differences were present for all the three markers. Two-tailed t-test: *Sox9*, not significant (n.s.) (*p* = 0.379); *Lgr5*, n.s. (*p* = 0.269); *Lgr6*, n.s. (*p* = 0.150).

In order to understand if the investigated HF stem cell markers could have a potential role as prognostic markers in these types of skin tumours of the *dog*, differences between benign and malignant tumours were analysed for both protein and mRNA expression. However, the majority of the malignant tumours included in the present work were epidermal tumours, resulting in bias in the distribution of malignant tumours in our case series. So, we limited the comparison to the TE group. A tendency towards an increase of *Sox9* and *Lgr6* mRNA expression in malignant TE was observed compared with benign cases (Figure 13). 

## 4. Discussion

In the present work, we investigated the expression of three putative HF stem cell markers in cutaneous epithelial tumours of dogs. Stem cell marker expression in neoplastic cells could have different meanings. They mark cancer stem cells that (a) can originate from tumour cells derived from the HF SCs and can represent the tumour cell of origin or (b) are neoplastic dedifferentiated cells that have acquired new stemness properties. Conditions (a) and (b) appear to be different, even opposite. However, based on new concepts of CSC plasticity and stem cell niche [2], we cannot exclude the possibility that the two situations can be simultaneously present in the heterogeneous population of a tumour. 

Interestingly, *Lgr*6 gene was highly expressed in the epidermal tumours analysed. It has been shown in *mice*, that Lgr6 protein expression is located in the stem cell niche above the bulge; this protein is considered as a marker of HF stem cells that are able to give rise to all lineages of the skin (HF, sebaceous gland and interfollicular epidermis), and to contribute to wound healing. In line with this, *Lgr6* was expressed in both HF and epidermal skin tumours of our study, indicating a potential common cell of origin of these tumours, that are then able to differentiate into different lineages. In accordance with our findings, Lgr6 was identified as an epithelial stem cell marker in mouse SCCs, as well [26]. This would suggest that, in dogs, similar to what has been hypothesized in *human* [27], the cell of origin of these epidermal tumours can be represented by: an interfollicular epidermal stem cell, re-expressing hair follicle stem cell markers; an hair follicle stem cell; or even a differentiated tumour cell that can be reprogrammed and expresses HF stem cell markers.

Lgr5 is a more specific HF stem cell marker compared with Lgr6 and Sox9, that is able to regenerate the cycling part of the HF and give rise to only the different HF structures, under normal conditions. Compatible with this is that its mRNA expression was observed only in HF tumours, but not in SCC and BCC. This is in line with previous studies on human SCC, where Lgr5 protein expression was not observed either [26]. In mice, however, in a specific skin condition determined in *Lgr6* germline KO *mice*, *Lgr5* gene expression could be upregulated in a compensatory mechanism, contributing to the development of cutaneous SCC [26,28]. Despite the high mRNA expression levels of all three SC markers, no immunolabelling was observed in any of the HF tumours investigated. Since the surrounding HFs were positively labelled by the antibodies, we have to conclude that the protein expression in the tumour is very low. This might be the consequence of post-translational regulation mechanisms, resulting in protein expression which is too low to be detectable with the applied immunohistochemical technique. 

The immunohistochemical expression pattern of Sox9 in canine normal HFs, as already previously reported by Gerhards et al. [11], would confirm it as a marker of stem cells already committed to ORS differentiation, as in human HFs [29]. We observed a Sox9-positive cell subpopulation in both epidermal and HF tumours, confirming results reported by Fantinato et al. [17]. Our results would indicate, in some canine HF tumours, such as IKA, TE and TB, as well as in SCC and BCC, the presence of ORS differentiation and/or a potential role of these cells in the development of these tumour types. These results are in agreement with previous results reported in *humans*, where Sox9 protein was expressed in BCC samples, as well as in TE and TL, indicating Sox9 as a general feature of differentiation of these types of tumours [30]. In our cases, as in *humans* [30], Sox9 immunostaining was almost completely restricted to tumour cells with basal cell-like morphology, and this was particularly evident in IKA and SCC positive cases.

Both Lgr5 and Lgr6, together with the other member of the same receptor family, namely Lgr4, are recognized mediators of the Wnt signalling pathway, that is activated in human HF tumours, especially TB [31], and probably in canine TB as well, as indicated by the presence of numerous positive nuclei [14]. In accordance with that, both *Lgr5* and *Lgr6* showed the highest mRNA expression levels in this tumour type in *dogs* (present results) and *humans* [19]. In both species, it is sometimes difficult to differentiate between BCC (epidermal origin) and TB (follicular origin) histologically, and a common origin of the two neoplasms has also been discussed [24]. Indeed, both neoplasms are composed of basaloid cells, with overlapping histopathological features [24,32]. This problem has been specifically addressed in numerous human published studies. Although some markers (such as AR, CK20 and PHLDA1) have been proposed as useful in distinguishing BCC from benign, hair-germ tumours (such as TB), no single marker seems to be completely sensitive or specific for this distinction [33]. The expression pattern of Sox9 has also been used to reiterate the follicular origin of human BCC by some authors [30,34]. Indeed, Sox9 expression, together with the cytokeratin profile of human BCC [35], would suggest a differentiation of these tumours along the ORS [34]. Comparable results on canine BCC obtained in the present study would also suggest a differentiation of some BCC cells along the ORS, similar to what we found in TB. However, recent results in *dogs* demonstrated a different cytokeratin (CK) and stem cell marker expression pattern profile in the two tumours: the basal cell markers p63 and CK14 were diffusely expressed in BCC, while TB tumours were strongly positive for CK8 and CD34, CK15 and CK19 [18]. Besides that, in the present study we observed statistically significant differences in the levels of mRNA expression of the three selected SC markers in the two tumours, suggesting their (in particular, Lgr5) use as potential markers in the distinction between BCC and TB. Taken together, the data would suggest that different neoplastic (stem cell) populations could be involved in the development/maintenance of these two neoplasms. However, it still remains unknown if the expression of these markers would indicate that the two tumours recognize different TICs, or if their neoplastic cells could dedifferentiate and acquire different stem cell marker expression. One possible hypothesis for human BCC [36] considered that adult interfollicular tumour-initiating/stem cells are reprogrammed into an embryonic hair follicle progenitor-like fate during BCC initiation. However, based on different experimental conditions, several adult somatic or stem cells of the skin can participate in tumour initiation [27], rendering difficult to understand the in vivo mechanisms of skin cancer development. 

We tried to investigate a possible role of the selected stem cell markers in cancer progression and their potential relation with histologically assessed tumour malignancy. Although differences were not significant, a trend towards upregulation of *Sox9* and *Lgr6* mRNA expression in malignant TE compared with benign ones was observed. This is in accordance with our previous studies on Sox9 immunohistochemical expression in different canine tumours types [4], including sebaceous gland tumours [37]. However, further studies should be performed to confirm the suggested potential role of Sox9 and Lgr6, both at the protein and mRNA level, as cancer markers of malignancy. 

## 5. Conclusions

In conclusion, we confirmed the expression of some putative HF stem cell markers in epidermal canine skin tumours, namely SCC and BCC, in the present study. Furthermore, we propose that the evaluation of these HF SC markers, and more specifically *Lgr5* mRNA expression, could be useful to determine the final diagnosis in ambiguous cases between TB and BCC. However, a future study on a larger case series is needed to further evaluate the discriminatory potential of Lgr5. Lastly, *Lgr6* and *Sox9* mRNA appeared to be more expressed in malignant forms of specific tumour types (TE), indicating a potential role of the two molecules in cancer progression and as prognostic markers. 

The present study, together with previous published work, indicate that the wide range of histomorphological differentiation of canine skin tumours is related to the heterogeneous stem cell population present in the skin, that could play the role of TICs, and to the tight communication of these cells with their niche. Understanding how normal/cancer stem cells are regulated and communicate with cells of the microenvironment, thus contributing to cancer development and/or progression and drug resistance mechanisms, will represent the future steps in this field of research. 

## Figures and Tables

**Table 1:** Expression level of Sox9 in the different tumours detected by immunohistochemistry (IHC).

Tumour Type	Total Number of Cases	Absent	Low 0%–10%	Moderate 10%–25%	High 25%–50%	Very High >50%	Overall Expression
SCC	9	5	0	3	1	0	5/9 absent
BCC	6	3	0	2	0	1	3/6 (50% of cases) absent
TL	3	1	1	1	0	0	variable (from absent to moderate)
IKA	9	2	2	3	1	1	5/9 moderate/low
TB	8	3	1	2	1	1	4/8 (50% of cases) absent/low
TE	11	5	4	1	1	0	9/11 absent/low
PM	6	1	3	1	0	1	4/6 absent/low

SCC: squamous cell carcinomas; BCC: basal cell carcinomas; TL: tricholemmomas; IKA: infundibular keratinizing acanthomas; TB: trichoblastomas; TE: trichoepitheliomas; PM: pilomatrichomas.

## Supplementary Materials

The following are available online at https://www.mdpi.com/2306-7381/7/2/62/s1, Table S1: RT-qPCR primers and probes, Table S2: Summary of the results obtained by IHC, RT-qPCR and histological analysis of the samples;
