# Supplementary material for: Canine Epithelial Skin Tumours: Expression of the Stem Cell Markers Lgr5, Lgr6 and Sox9 in Light of New Cancer Stem Cell Theories
Source: Vet Sci. 2020 May 8;7(2):62. doi: 10.3390/vetsci7020062 (PMC7356500; doi:10.3390/vetsci7020062)
Supplement: Supplementary file 1 [file vetsci-07-00062-s001.pdf]

Supplementary Materials

Supplementary table 1. RT-qPCR primers and probes.

Candidate gene	Forward primer	Reverse primer	Probe	Amplicon length	Reference sequence
Sox9	TTCCGCGACGTGG ACAT	TCGAATTCGTTGACGT CGAA	AGCAGCGACGTCATCT CCAACATAGAGAC	77bp	NM_00100 2978.1
Lgr5	GGCTCCACAGCCT AGAGACTTTAG	TTGTTGCTGTGAAATC CTAGTTCTTT	AATCTTGATGAATTCC CCACCGCCA	109bp	XM_846738 .2
Lgr6	CTGGGCAGACTGC AGGAACT	GCAGAGGGTTCCCCAT GAAA	AACAACATCAAGGCC ATCCCAGAGAAGG	82bp	XM_003562 2187.1

Supplementary table 2. Summary of the results obtained by IHC, RT-qPCR and histological analysis of the samples. .

	IHC	RT-qPCR			Histological analysis (H&E staining)						
	Sox9	Sox9	Lgr5	Lgr6	Differentiation	Invasion	Ulceration/ haemorragies	Mitosis	Apoptosis	Necrosis	Inflammation
**SCC**											
1	0	0.03	0.02	0.09	WD	Yes (focal)	no	21/17	+++	yes	2-3; L, PC
2	0	0.03	0.07	0.09	WD, in situ	No	yes	3/10	+	No	2; N, due to ulceration
3	0	0.05	0.01	2.52	MD/PD	yes	yes	13/10	+	yes	2; N
4	0	0.06	0.02	0.36	PD	yes	Yes	16/10	+++	yes	2; N
5	0	0.14	0	0.69	WD	yes	yes	16/10	+	yes	2; L, PC
6	25>-50	0.15	0.01	0.65	PD	yes	yes	28/10	+++	yes	3; N, L, PC
7	10>-25	10.25	0.27	0.11	PD/spindle cell	yes	yes	32/10	+	yes	4; N, L, PC
8	10>-25	74.25	0	0	PD	yes	yes	18/10	+++	yes	3; N, L, PC
9	10>-25	0.28	0.18	0.74	WD	Yes	Yes	12/10	++	No	1; L, PC, N
**BCC**											
1	10>-25	0.23	0	0.34	Basosquamous	Yes	Yes	28/8	-/+	Yes	1; L, PC, N
2	0	0.13	0.06	5.18	Scattered foci of squamous differentiation	Yes	No	26/10	++	Yes	1; L, PC
3	0	0.03	0	0.04	Basal	Yes	Yes	19/10	++	Yes	1; L, PC, N
4	0	0.14	0.01	0.53	Scattered foci of squamous differentiation	No	No	3/10	+	No	1; L, PC
5	25>-50	0.29	0.01	9.60	Basal	Yes	Yes	64/10	-	Yes	1; N
6	>50	0.32	0.06	2.03	Basal, some parts with vacuolated cytoplasm	Yes	Yes	26/10		Yes (+++center)	2; L, PC, N
**TL**											
1	0	0.68	5.14	2.91	Bulb type	No	No	4/10	-/+	No	0; scattered L
2	10>-25	1.65	1.98	25.55	Bulb type	No	No	10/10	-/+	No	0
3	0>-10	0.53	0	0.01	Bulb type	Yes	No	3/10	-/+	No	0; scattered L
**IKA**											
1	0	3	0	0.49	WD, MALIGNANT	Yes	Yes	5/10	+	Yes	4; N, L, PC
2	0	0.40	0.06	0.38	WD, MALIGNANT	Yes	Yes	8/10	++	Yes	4; N, L, PC
3	10>-25	0.55	0.11	0.48	WD	No	No	5/10	-/+	No	2; L, PC (surrounding the neoplasm)
4	10>-25	1.03	0.58	0.22	WD	No	No	5/10	-/+	No	No
5	25>-50	1.20	0.79	0.24	WD	No	No	5/10	-/+	No	No
6	>50	1.09	4.98	3.84	WD	No	No	5/10	-/+	No	No
7	0>-10	1.37	7.22	4.42	WD	No	No	5/10	-/+	No	No
8	0>-10	2.32	2.04	0.63	WD	No	No	5/10	-/+	No	2; L, PC (within the neoplasm)
9	10>-25	0.65	0.36	0.77	WD	No	No	5/10	-/+	No	1; L, PC (within the neoplasm)
**TB**											
1	10>-25	0.89	8.99	20.52	Ribbon type	No	Yes	112/10	-/+	No	2; N
2	25>-50	0.81	1.72	6.6	Ribbon type	No	No	33/11	+	No	No
3	25>50	3.60	1.37	66.04	Ribbon type	No	No	20/10	-/+	No	No

4	0	1.79	0.29	25.96	With ORS diff	No	Yes	16/10	++	No	No
5	0	19.24	0.86	46.94	Trabecular/papillar/spindle, with cavernous cyst	No	No	21/11	-	No	No
6	10>-25	0.24	8.04	3.13	With ORS diff, spindle	No	No	21/10	++	No	No
7	0	1.16	9.74	11.79	Trabecular type	No	No	23/8	+	No	No
8	0>-10	1.40	5.83	13.28	Trabecular type. and papillary bodies?	No	No	62/10	-	No	No
**TE**											
1	0>-10	1.18	0.04	0.39	Infundibular cyst, matrical cords and nests	No	No	48/10 (matrical)	-/+	No	2; L, mainly perivascular in the surrounding dermis
2	0>-10	0.19	0.05	0.4	Mainly matrical	No	No	65/10	-	No	No
3	0	0.60	0.01	0.14	Mainly Infundibular	No	No	1/10	-/+	No	No
4	0	0.23	1.04	0.99	Mainly matrical	No	No	26/10	-/+	No	1; L, surrounding cystic structures
5	0	0.22	0.88	0.61	Mainly Infundibular/Basal cell	No	yes	115/10	-	No	1; L, PC
6	10>-25	0.21	0.88	2.94	MALIGNANT TE Mainly matrical with intermediate diff	Yes	yes	50/10	+	No	2; L, PC
7	25>-50	8.49	0.45	6.15	MALIGNANT TE Mainly matrical with intermediate diff	Yes	No: ulceration; Yes: haemorrages	40/10	+	Yes	3; L, PC, associated with invasive nests
8	0	5.86	0.07	3.31	MALIGNANT TE Basosquamous	Yes	Yes	79/8	++	Yes	3; L, PC
9	0	0.27	2.46	2.24	MALIGNANT TE Mainlymatrical with intermediate diff	Yes	Yes	81/10	++	Yes	3; L, PC, with associated pyogranuloma
10	0	0.27	0.25	0.07	MALIGNANT TE Mainly matrical with foci of IRS diff.	No	No	80/10	-	No	2; L (aggregates)
11	0	0.12	0.66	0.3	MALIGNANT TE Mainly matrical with intermediate diff	Yes focal	Yes	39/10	-/+	Yes	3; L, PC, with associated pyogranulomas
**PM**											
1	10>-25	0.14	0.20	1.69	Multiple foci of squamous diff	Yes	No	77/10	+	Yes	2; Granulomatous
2	0>-10	0.49	3.23	1.98	IRS* diff in one area of the wall (but trichoyaline granules not visible)	No fibrous tissue surrounding	No	50/10	-/+	No	1; L, PC, mainly perivascular
3	0>-10	0.20	1.62	4.25	Multifocal IRS* diff within the wall. Rare squamous diff	No, dome shaped	No	51/10	-/+	No	2; L, PC
4	0>-10	0.18	0	0.20	Multifocal IRS* diff within the wall.	No	No	89/10	+++	Yes central	2; L, PC mainly surrounding the neoplasm
5	0	0.16	0	0.01	Multifocal IRS* diff within the wall.	No	No	64/10	++	Yes central	2; L, PC mainly surrounding the neoplasm
6	>50	0.34	0.49	0.70	cystic	No	No	13/10	+	No	1-2; L, PC surrounding cystic structure

IHC: immunohistochemistry; H&E: hematoxylin and eosin staining. SCC: squamous cell carcinoma; BCC: basal cell carcinoma; IKA, infundibular keratinizing acanthoma; PM, pilomatricoma; TB, trichoblastoma; TE, trichoepithelioma; and TL, tricholemmoma. IRS: inner root sheath; ORS: outer root sheath. Diff.: differentiation. WD: well differentiated; MD: moderately differentiated; PD: poorly differentiated. Inflammatory cells: L: lymphocytes; PC: plasma cells; N: neutrophils. Grade of inflammation: 0: none/minimal; 1: mild; 2: moderate; 3: severe; 4: marked. Apoptosis score applied:

-: none; −/+: scattered, rare apoptotic cells (<5%); +: about 5%; ++: 6–10%; +++: more than 10% of apoptotic cells in a 20X field (evaluated in 10 20× fields). IHC results: for each sample, Sox9 positive cells were analyzed in 10 HPF (40×) and based on the protein expression levels 5 ranges were made: absent (0): no positive cells; low: >0%–<10% positive cells; moderate: ≥10%–<25% positive cells; high: ≥25%–<50% positive cells; very high: ≥50% positive cells. RT-qPCR results: Reactions were performed in triplicate and relative amount of cDNA was normalized to a reference gene. Fold changes were calculated based on expression in normal skin (n = 3).

Supplementary file 1. Immunofluorescence protocol.

Sections were deparaffinized and rehydrated by passage through xylene and graded ethanols. Antigen retrieval was carried out in either Sodium Citrate buffer (self made) pH 6.0 for 20 min at 80 °C or in TrisEDTA buffer (self made) pH 9.0 for 15 min in a pressure cooker. Non-specific background was blocked using 5% dried skim milk in PBS. Subsequently, primary anti-Lgr6 antibody were diluted in Dako REAL™ Antibody Diluent (S2022, Dako, Baar, Switzerland) and applied for 60 min at room temperature or overnight +4 °C. After primary antibody incubation, slides were incubated with Alexa fluor conjugated goat anti-rabbit secondary antibody (1:200) (SigmaAldrich, St. Louis, MO, USA) for 30 min at room temprerature. After washing steps, slides were incubated for 2 min with DAPI solution (SigmaAldrich, St. Louis, MO, USA) at room temperature and then mounted and visualized in a fluorescence microscope. No immunofluorescence was detected in any of the experiments performed, neither in the tested samples nor in the included positive controls (human normal skin and canine trichoblastoma).
